# Cortical Neural Computation by Discrete Results Hypothesis

**DOI:** 10.3389/fncir.2016.00081

**Published:** 2016-10-19

**Authors:** Carlos Castejon, Angel Nuñez

**Affiliations:** Department of Anatomy, Histology and Neuroscience, School of Medicine, Autonomous University of MadridMadrid, Spain

**Keywords:** cerebral cortex, sensory processing, cell ensembles, fast-spiking cells, brain oscillations, discrete computation, neural synchronization, processing resolution

## Abstract

One of the most challenging problems we face in neuroscience is to understand how the cortex performs computations. There is increasing evidence that the power of the cortical processing is produced by populations of neurons forming dynamic neuronal ensembles. Theoretical proposals and multineuronal experimental studies have revealed that ensembles of neurons can form emergent functional units. However, how these ensembles are implicated in cortical computations is still a mystery. Although cell ensembles have been associated with brain rhythms, the functional interaction remains largely unclear. It is still unknown how spatially distributed neuronal activity can be temporally integrated to contribute to cortical computations. A theoretical explanation integrating spatial and temporal aspects of cortical processing is still lacking. In this Hypothesis and Theory article, we propose a new functional theoretical framework to explain the computational roles of these ensembles in cortical processing. We suggest that complex neural computations underlying cortical processing could be temporally discrete and that sensory information would need to be quantized to be computed by the cerebral cortex. Accordingly, we propose that cortical processing is produced by the computation of discrete spatio-temporal functional units that we have called “Discrete Results” (Discrete Results Hypothesis). This hypothesis represents a novel functional mechanism by which information processing is computed in the cortex. Furthermore, we propose that precise dynamic sequences of “Discrete Results” is the mechanism used by the cortex to extract, code, memorize and transmit neural information. The novel “Discrete Results” concept has the ability to match the spatial and temporal aspects of cortical processing. We discuss the possible neural underpinnings of these functional computational units and describe the empirical evidence supporting our hypothesis. We propose that fast-spiking (FS) interneuron may be a key element in our hypothesis providing the basis for this computation.

## Introduction

The cerebral cortex is possibly one of the most complex natural systems. Untangling its intricate functional microcircuit is one of the formidable challenges of neuroscience. However, despite its importance, how cortical computations are performed and the underlying neural mechanisms remain unclear.

There is increasing evidence that the power of the cortical processing is produced by populations of neurons forming dynamic neuronal ensembles. Theoretical proposals (Lorente de Nó, 1938; Hebb, 1949; Hopfield, 1982; Engel et al., 2001; Buzsáki, 2010; Yuste, 2015) and multineuronal experimental studies (Fujisawa et al., 2008; Miller et al., 2014) have revealed that ensembles of neurons can form emergent functional units. However, it is still unknown how distributed neuronal activity can be functionally integrated to contribute to cortical computations. Moreover, no one knows what these functional units look like, or how they emerge. In sum, how these ensembles are implicated in cortical computations is still a mystery.

Although cell ensembles have been associated with brain rhythms, the functional interaction remains largely unclear. It is still unknown how spatially distributed neuronal activity can be temporally integrated to contribute to cortical computations. A theoretical explanation integrating spatial and temporal aspects of cortical processing is still lacking.

In this Hypothesis and Theory article, we propose a new functional theoretical framework to explain the computational roles of these ensembles in cortical processing. We suggest that complex neural computations underlying cortical processing could be temporally discrete. Accordingly, we propose that cortical processing is produced by the computation of discrete spatio-temporal functional units that we have called “Discrete Results” (Discrete Results Hypothesis). Furthermore, we propose that precise dynamic sequences of Discrete Results is the mechanism used by the cortex to extract, code, memorize and transmit neural information.

As we describe in the next sections, this proposal has the ability to match the spatial and temporal aspects of cortical processing. Moreover, our hypothesis represents a novel functional mechanism by which information processing is computed in the cortex. We discuss the possible neural underpinnings of this proposal and describe the empirical evidence supporting our hypothesis.

## Neuronal Processing by Distributed Neuronal Activity

The power of the cortex lies in the dynamic coordination of neurons (Hebb, 1949; Pouget et al., 2000; Yuste, 2015). Coordinated activity of large ensembles of spatially distributed cells across the cortex provides the source for the processing and encoding of sensory information. Experimental work supports this idea. Highly distributed representations of tactile information have been described in the cortex (Nicolelis et al., 1997). In the visual cortex, sensory stimuli recruit intrinsically generated cortical ensembles (Miller et al., 2014; Okun et al., 2015). Representation of motor programs via cell ensembles has been described (Hommel, 2004). Moreover, the auditory cortex is dominated by broad scale dynamics in which a complete representation of sounds emerges only at a global scale (Bathellier et al., 2012).

Accordingly, multineuronal recording studies (Fujisawa et al., 2008; Miller et al., 2014) have revealed that ensembles of neurons can form emergent functional units. Therefore, they may be the building blocks used in cortical processing. However, important questions concerning integration of cortical activity remain unresolved. It is still unknown how these ensembles are present in the cortex and how spatially distributed cells can functionally contribute to unified stimulus codification. Moreover, although cell ensembles have long been thought to be associated with brain rhythms (Harris et al., 2003), a theoretical explanation integrating spatial and temporal aspects of cortical processing has yet to be proposed.

## Rhythmic Neuronal Activity in the Brain

Mammalian brain activity is rich in rhythms. The preservation of that rhythmic activity during the course of evolution demonstrates its relevance and appears to reflect a common functional mechanism for neural processing (Buzsáki and Draguhn, 2004). These rhythms are involved in perception, attention, memory, consciousness and movement execution (Gray et al., 1989; Engel et al., 2001; Fries et al., 2001; Brown, 2007; Buschman and Miller, 2010; Baldauf and Desimone, 2014). They play a key role in neural communication (Fries, 2005; Schroeder and Lakatos, 2009; Siegel et al., 2012). Moreover, it has been suggested that oscillatory activity contributes to spike synchronization of distributed neurons (Gray et al., 1989; Nuñez et al., 1992; Engel et al., 2001) and that synchrony underlies binding of separate features enabling perceptual unity (Singer and Gray, 1995).

Excitation and inhibition play a key role in the generation of rhythmic activity (Steriade et al., 1993; Whittington et al., 1995; Traub et al., 1996; Cardin et al., 2009). However, the mechanisms underlying oscillations and synchrony are still not well understood. Furthermore, current theories about their computational role are incomplete (Thiele and Stoner, 2003; Roelfsema et al., 2004; Hermes et al., 2015). It remains unclear what function they play in neural processing. They may contribute to discretize the computation.

## Discrete Neural Computation

**Figure 1:**
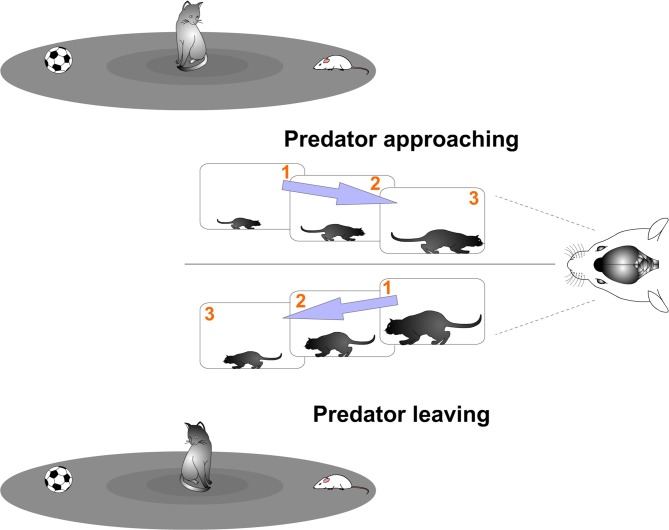
**Discrete neural processing.** Successful interaction with the world depends on accurate processing of rapidly changing stimuli. It requires a precise detection of changes sampling the flow of sensory information to compare its contents. Of particular interest are relevant events, such as the appearance of a predator. A challenge faced by the prey’s brain is to rapidly extract predator changing features from the scene in order to react satisfactorily. In this hypothetical example, the prey’s visual system actively samples the visual environment. Discrete samples are taken separately by brief temporal periods. Predator movements (choosing between the prey and the ball) can be detected by computing differences between the subsamples (see “Cortical Neural Computation by Discrete Results Hypothesis: Functional Spatio-Temporal Units of Computation” Section). That computation allows the prey to know if the predator is approaching or leaving and to take appropriate action.

The brain receives a constant flow of analog sensory information from the environment. Successful interaction with the world depends on accurate processing of that information. Therefore, the challenging task that the brain faces is to rapidly extract changing relevant features from the environment in order to respond adequately. It requires a precise detection of changes sampling the flow of sensory information to compare its contents. Consequently, a primary function of the brain could be to discretize the continuous flow of information, compare those sampled units and extract relevant information from that computation (Figure 1). In sum, although the brain receives a constant flow of analog sensory information from the environment, sensory stimuli changes will be sampled in a discrete manner.

Scientists have long theorized that our cognition operates discontinuously within a framework of discrete cycles (Pitts and McCulloch, 1947; Harter, 1967; Allport, 1968; Varela et al., 1981; VanRullen and Koch, 2003; Buschman and Miller, 2010). Accordingly, neural systems could undergo oscillatory activity patterns. These oscillations could divide the neural processing into a series of discrete computational events.

There are relevant experimental data demonstrating this discrete processing. Discrete computations are well described in visual perception (VanRullen et al., 2005). One example is microsaccadic eye movements by which the visual system acquires fine spatial detail (Ko et al., 2010). Accordingly, vision is interrupted and sensory processing is discretized, separating it into distinct epochs. Recent evidence has been shown for discrete perceptual sampling in the somatosensory domain (Baumgarten et al., 2015). They demonstrated that somatosensory perception operates in a discrete mode, with sensory input being sampled by discrete perceptual cycles. Memory (Lundqvist et al., 2016) and attention are other examples of this computational nature (Buschman and Miller, 2009; Busch and VanRullen, 2010). It is known that oscillatory neuronal activity in the frontal eye field reflects the successive cycles of a sequential attentional exploration process during visual search (Buschman and Miller, 2009). Furthermore, discretized processing and encoding in the hippocampus is well described (Buzsáki, 2005). However, how the brain, especially the cerebral cortex, performs this computation is unknown.

## Cortical Neural Computation by Discrete Results Hypothesis: Functional Spatio-Temporal Units of Computation

One of the most challenging problems we face in neuroscience is to understand how the cortex performs computations. Here we suggest that complex neural computations underlying cortical processing could be temporally discrete. But how does the cortex perform this computation? We propose that cortical processing is produced by the computation of discrete emergent functional units that we have called Discrete Results (Discrete Results Hypothesis). As we describe in the next sections, this novel concept has the ability to match the spatial and temporal aspects of cortical processing. We discuss the possible neural underpinnings of these spatio-temporal computational units and describe the empirical evidence supporting our hypothesis.

Our Discrete Results Hypothesis suggests that the computational principle of the cortex lies in the precise temporal coordination of spikes of spatially distributed neurons. It is necessary to divide the temporal and spatial dimension of that proposal for a better clarification.

### Spatial Dimension of Cortical Processing: The “Ensemble” as a Functional Spatial Unit

In the cortex, most neuronal activity occurs in the form of coactive groups of cells defining neuronal ensembles (Miller et al., 2014). However, it is unclear how they emerge, with which neurons, what and how the relation is between the members, what spatial and temporal extension they have and what exactly an ensemble functionally means.

**Figure 2:**
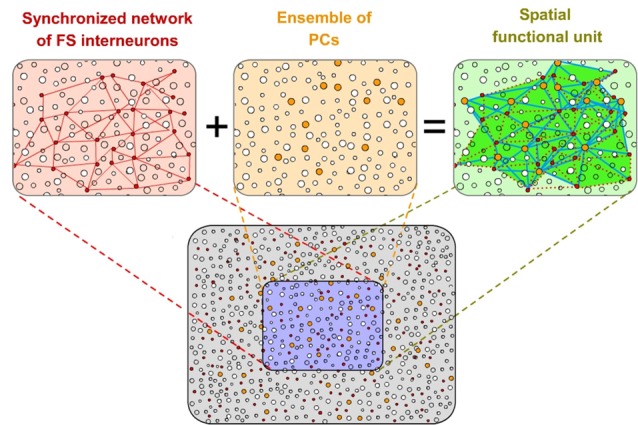
**Spatial functional units of cortical processing.** A specific spatially distributed set of pyramidal cells (PCs; in orange) that are controlled by a specific coupled network of fast-spiking (FS) inhibitory cells (in red) form functional spatial units (“Ensembles”) of cortical computation (in green). All PCs organized by that particular inhibitory network form part of that Ensemble. Individual PCs could participate in different Ensembles. The members and spatial extension of the Ensemble is determined by the inhibitory network. Red lines represent mutual connections between FS inhibitory cells in the network. These neurons innervate strategically the PCs (blue lines) extending a blanket of precise inhibition onto them.

In our hypothesis, we define “Ensemble” as a specific spatially distributed set of excitatory neurons (referred to as pyramidal cells hereafter, PCs) that are controlled by a definite synchronized network of fast-spiking (FS) inhibitory cells (see “Neural Underpinnings of Discrete Results Hypothesis : Spatio-Temporal Integration By Fast-Spiking Cells Synchronized Network.” Section). All PCs organized by that particular synchronized inhibitory network form part of that Ensemble. It means that the Ensemble is formed by all the PCs whose firing could be transiently constrained by that specific synchronized inhibitory network (Figure 2). The members and spatial extension of the Ensemble is determined by that inhibitory network. Moreover, individual PCs could participate in different emergent Ensembles.

These emergent clusters of PCs form functional spatial units of cortical computation. However, that spatial aspect must be complemented with a temporal one.

### Temporal Dimension of Cortical Processing: “Temporal Structure of Spikes”

Neurons are temporally precise on very fine timescales (Mainen and Sejnowski, 1995; Shmiel et al., 2005; Butts et al., 2007). Experimental evidence indicates that the exact time point at which a spike occurs plays an important role in information processing (Markram et al., 1997). Moreover, the precise timing of neuronal spiking is vital for coding of information (Singer and Gray, 1995). Therefore, this temporal precision is likely to be crucial for cortical computation. However, the functional significance remains unclear.

**Figure 3:**
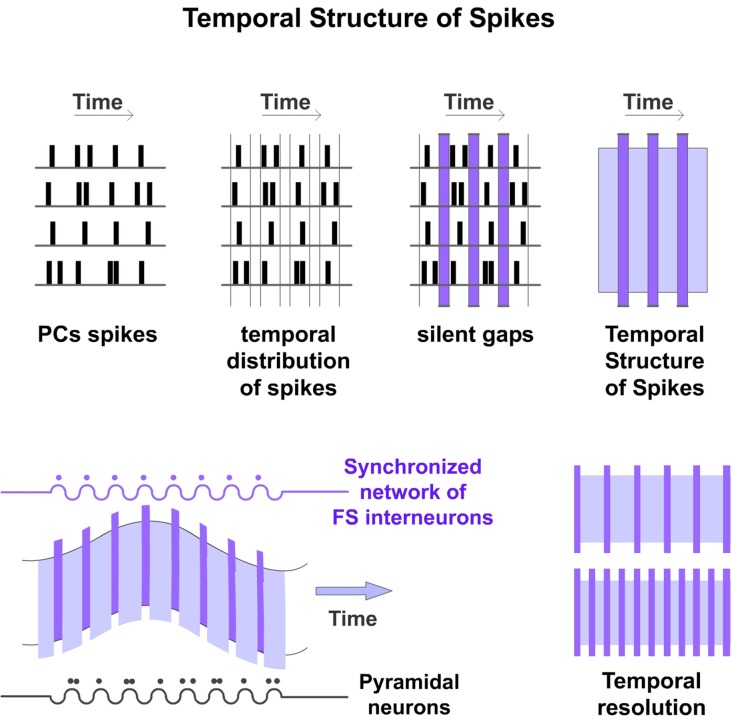
**Temporal structure of spikes.** Spike timing of PCs is constrained by FS inhibitory interneurons. These cells set the timing and rate of action potentials produced by PCs limiting the temporal window during which they can be generated (Pouille and Scanziani, 2001). Moreover, PCs cannot discharge when they are shunted by strong inhibition. Consequently, the synchronized spiking of the inhibitory network creates temporal order among the PCs firing, generating precise silent periods that we have called “Silent Gaps” during which action potentials cannot be generated (vertical columns in purple). Rhythmic inhibition of the interneuron network provides alternating periods of spiking and no spiking in the Ensemble of PCs constraining these spikes into discrete periods generating a scaffold that we have called “Temporal Structure of Spikes”. Rows represent hypothetical spike rasters for an Ensemble of four PCs. Spikes from these PCs occur independently but organized inside that temporal structure (top). These cycles would result in the oscillations observed in the brain (bottom left). The temporal structure can be dynamically adjusted to meet the finest processing resolution depending on perceptual, task or attentional demands (bottom right).

The Discrete Results hypothesis suggests that cortical processing is produced by a highly ordered temporal organization. Spike timing of PCs in a particular Ensemble is constrained by an inhibitory network generating a precise structured firing. We propose that this network constrains PCs spikes in temporal precise manner creating what we have called “Temporal Structure of Spikes” (Figure 3). We defined it as the accurate spike timing organization resulting from the precise temporal suppression of PCs spikes in the Ensemble. Spikes from PCs occur independently but organized inside the temporal structure. We propose that this Temporal Structure of Spikes is very important in the processing, coding and transfer of information in the cerebral cortex. The temporally structured firing activity enables information to be processed and coded in a way that downstream networks can compute. Accordingly, the existence of this specific temporal structure implies that failures in that precision of spikes will result in processing dysfunctions. An example of the importance of the temporal structure is the spike-timing-dependent plasticity (Caporale and Dan, 2008). This temporal structure is not fixed. It can be dynamically adjusted (for example by sensory input or by top-down influence) to meet the finest processing resolution depending on perceptual, task or attentional demands. Furthermore, the structure could be adjusted by neuromodulators. Accordingly, variations in the temporal structure will produce changes in the rate and temporal precision of PCs firing in the Ensemble. PCs spikes latencies and synchronization between them will vary accordingly. Consequently, changes in that temporal precision will code different content.

Experimental data provide support for this proposal. It is known that spike timing of PCs is constrained by the inhibitory cells. FS interneurons quickly limit the temporal window during which action potentials can be generated (Pouille and Scanziani, 2001; Li et al., 2015). Consequently, PCs are more likely to fire at precise points in time (Cardin et al., 2009). More importantly, PCs cannot discharge when they are shunted by strong inhibition. We propose that these precise inhibitory inputs to PCs generate strict periods of no spiking (“Silent Gaps”) in the Ensemble. Our hypothesis suggests that these precise Silent Gaps are very important for cortical computation. They divide the neural processing in the Ensemble into a series of discrete computational events that we have called “Discrete Results”.

It is also known that FS interneurons generate synchronized networks by mutual chemical and electrical connections in the neocortex (Whittington et al., 1995; Traub et al., 1996; Galarreta and Hestrin, 1999; Gibson et al., 1999). Electrical synapses generate highly precise transmission between interneurons of these networks. We propose that this coupling promotes the harmonized firing of connected neurons (Jones et al., 2000; Deans et al., 2001; Bartos et al., 2007) forming a synchronized network imposing a time-dependent spike restriction in the Ensemble of PCs. Different synchronized networks of FS interneurons create different sets of possible Ensembles. The simultaneous firing of the FS interneurons in the inhibitory network generates a synchronized inhibitory activity at their postsynaptic PCs in the Ensemble. The synchronized spiking of the inhibitory network could be fast enough to adjust the onset spiking of PCs (Woodruff et al., 2011; Li et al., 2015) and to create a temporal structure or scaffold providing alternating windows of no spiking in the emergent Ensemble of PCs. The rhythmic functioning of this network creates a sequence of temporal discrete events. This network rhythmically concentrates PCs discharges to particular discrete moments providing observable oscillation cycles at population level.

In sum, the inhibitory network forms a spatial structure of synchronized FS inhibitory cells and then this synchronized network generates a temporal structure of firing in the Ensemble.

## Discrete Result: A Functional Spatio-Temporal Unit of Computation

**Figure 4:**
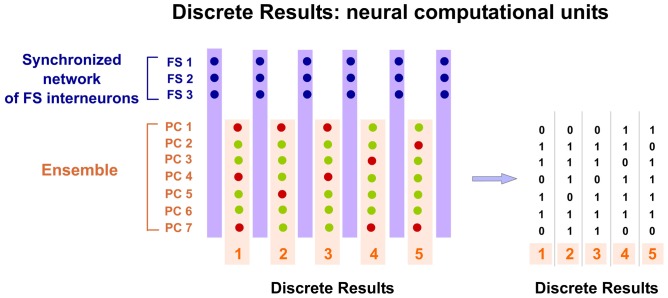
**Discrete Results: neural computational units.** PCs belonging to the same Ensemble participate in the Discrete Result. Therefore, all PCs in the Ensemble have the opportunity to fire. Spikes from these PCs occur independently but organized inside the temporal structure. Each potential Discrete Result emerges transiently formed by the combination of the firing and silent responses (no firing) of all PCs in the Ensemble. Therefore, PCs silent responses are also important in cortical computation and codification. Moreover, the same Ensemble can form multiple Discrete Results. This means that the same PCs of the Ensemble process and encode multitude of contents. Consequently, there are great potential neural possibilities that could form a Discrete Result and also enormous possibilities that could form different sequences of Discrete Results. In this hypothetical example, the Ensemble is formed by seven PCs and the synchronized network of FS interneurons by three cells. Five possible Discrete Results are shown. PCs spikes are displayed as green dots and silent responses (no spikes) as red dots. On the right, a digital (binary) representation of these hypothetical Discrete Results is shown.

Spatio-temporal activity patterns play an important role in cortical mechanisms of information processing (Ayzenshtat et al., 2010). Consequently, we propose that PCs compute and communicate information by using specific spatio-temporal patterns of spiking. Our hypothesis suggests that the cortex generates and employs these precise patterns to perform its computations. Thus cortical processing depends on the precise temporally structured relations among the respective spikes of PCs of the Ensemble. Information is encoded in the precise relations between temporal structured discharges. Individual spikes of PCs in the Ensemble take functional relevance when inserted into that temporal structure, forming a Discrete Result. Precise silent periods (Silent Gaps) inside the structure discretize the processing and allow for the formation of these discrete spatio-temporal functional units. All PCs belonging to the same Ensemble participate in the Discrete Result. Therefore, all PCs in the Ensemble have the opportunity to fire. Spikes from these PCs occur independently but organized inside the temporal structure. Each Discrete Result emerges transiently formed by the combination of the firing and silent responses (no firing) of all PCs in the Ensemble (Figure 4). Thus, PCs silent responses are also important in cortical computation and codification. The same Ensemble can form multiple Discrete Results. This means that the same PCs of the Ensemble, process and encode multitude of contents. Consequently, there are great potential neural possibilities that could form a Discrete Result and also enormous possibilities that could form different sequences of Discrete Results. Therefore, the number of possible representations that can be formed is titanic. This mechanism could explain why the cortex is so robust to damage. Moreover, the content coded by a Discrete Result depends also on the resolution of the Temporal Structure of Spikes.

**Figure 5:**
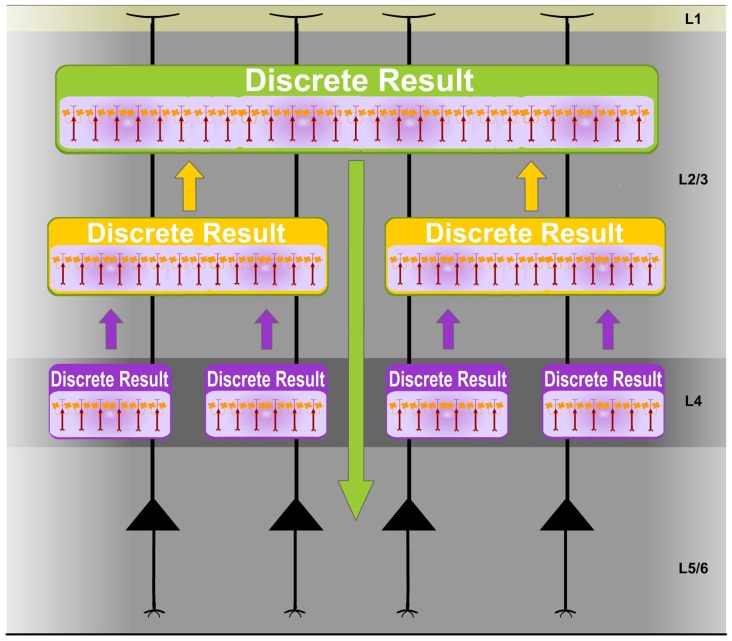
**Discrete Results: functional units of neural computational integration.** Different sets of possible Ensembles are created by different synchronized networks of FS cells along the cortical processing hierarchy. Experimental studies support this idea. Distinct clusters of FS interneurons have been identified in the cortex. For example, in the rat barrel cortex, one layer 4 FS interneuron type has an axonal domain strictly confined to a barrel (Koelbl et al., 2015). Accordingly, the cortex performs computations using multiple Ensembles in parallel creating a multitude of Discrete Results simultaneously. Discrete Results at higher levels integrate computational results from previous stages. Therefore, each Discrete Result constitutes a functional unit that has the ability to process, integrate and represent specific content (Discrete Results) from previous computations. Consequently, in sensory processing, they functionally contribute to unified stimulus codification. Therefore, the Discrete Result concept could explain the binding of separate features enabling perceptual unity. Experimental data provide support for this proposal. Highly distributed representations of tactile information have been described in the cortex (Nicolelis et al., 1997). Moreover, the auditory cortex is dominated by broad scale dynamics in which a complete representation of sounds emerges only at a global scale (Bathellier et al., 2012).

Individual PCs could participate in different Ensembles and be potentially implicated in multiple representations. Furthermore, different synchronized networks of FS interneurons create different sets of possible Ensembles. Accordingly, the cortex performs computations using multiple Ensembles in parallel creating a multitude of Discrete Results simultaneously. Moreover, different sets of possible Ensembles are created by different synchronized networks of FS interneurons along the cortical processing hierarchy. Discrete Results at higher levels integrate computational results from previous stages. Therefore, each Discrete Result constitutes a functional unit that has the ability to process, integrate and represent specific content (Discrete Results) from previous computations (Figure 5). Consequently, in sensory processing, they functionally contribute to unified stimulus codification.

**Figure 6:**
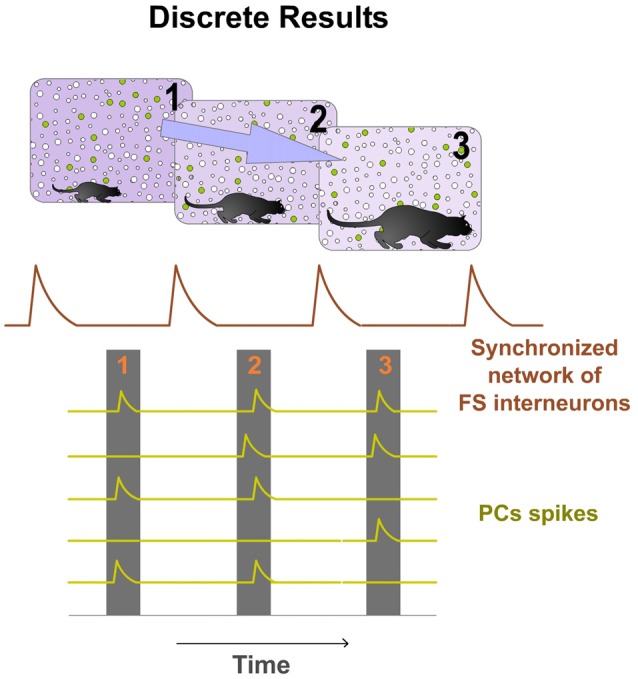
**Discrete Results in cortical sensory processing.** Hypothetical spatial maps of cortical neurons for each computational event resulting in quantization of the continuous flow of sensory information are shown. Green cells show a representative Ensemble of PCs organized by a specific synchronized network of FS interneurons. Individual spikes of these PCs take functional relevance inserted into a temporal structure forming discrete spatiotemporal functional units (Discrete Results). In this hypothetical example, relevant sensory information (predator movements) can be extracted by computing differences between the Discrete Results. Increasing the number of Discrete Results per temporal unit allows resolution enhancement.

The Discrete Result concept has the ability to explain how complex neural computations underlying cortical processing could be temporally discrete. Consequently, we propose that sensory information would need to be quantized to be computed by the cerebral cortex. Therefore, processing of sensory information must be temporally discrete and information flow in the cortex must be quantized allowing for the formation of Discrete Results. Therefore, in sensory processing, they can be defined as each neural computational functional unit resulting in quantization of the continuous flow of sensory information (Figure 6). Increasing the number of Discrete Results per temporal unit allows resolution enhancement. It could be dynamically adjusted by sensory input or by top-down influence to meet the finest processing resolution depending on perceptual, task or attentional demands.

## Neural Computation by Dynamic Sequence of Discrete Results

Multineuronal activity structured in temporal sequences has been suggested since long ago (Lorente de Nó, 1938; Hebb, 1949; Abeles, 1991). Experimental studies have increased our knowledge about how this sequential activity is generated in the brain (Harris et al., 2003). However, untangling its functional computational significance is still a formidable challenge today.

We propose that precise sequences of Discrete Results are the mechanism used by the cortex to perform computations. The computation of the Discrete Results sequence is the mechanism used by the cortex to extract, code, memorize and transmit neural information. This proposal is a neuronal population mechanism to compute and code. Dynamic sequences of Discrete Results generate representations. Different sequences codify different contents.

**Figure 7:**
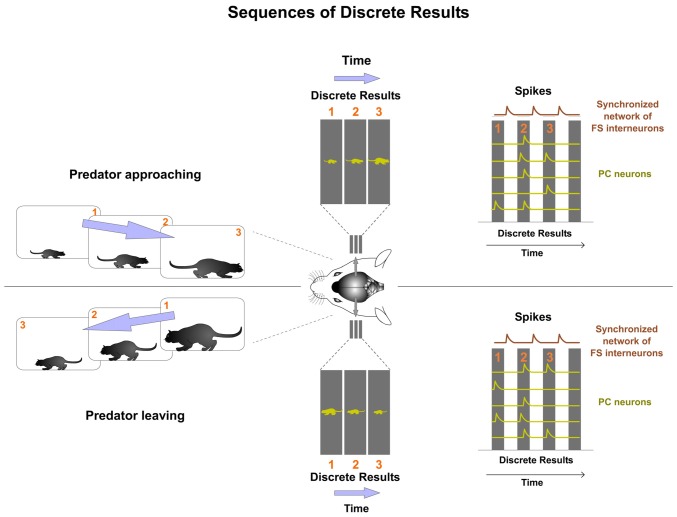
**Dynamic sequences of Discrete Results are the mechanism used by the cortex to perform computations.** Different sequences of Discrete Results codify different content. In this example, both types of predator trajectories involve different sequentially activated Discrete Results. One sequence codifies the predator approaching and the other leaving. By computing the successive Discrete Results in the sequence the prey’s cortex is allowed to extract predator trajectory in order to respond appropriately.

The rhythmic functioning of the synchronized inhibitory network creates a sequence of Discrete Results (Figure 7). Computations between successive Discrete Results in the sequence produce the power of the cortical processing. Experimental data provide support for this hypothesis. Sequential activity of multineuronal spiking has been well described in the cortex (Fujisawa et al., 2008; Crowe et al., 2010; Harvey et al., 2012; Carrillo-Reid et al., 2015) and in the hippocampus (O’Keefe and Burgess, 1996; Fyhn et al., 2004; Foster and Wilson, 2006; Pastalkova et al., 2008; Wikenheiser and Redish, 2015).

Moreover, cortical processing by dynamic sequences of Discrete Results could be the neural source of some rhythmic signals observed at population level. This hypothesis of neural processing could be applied to other structures and nuclei of the brain.

## Neural Underpinnings of Discrete Results Hypothesis: Spatio-Temporal Integration by Fast-Spiking Cells Synchronized Network

Our Discrete Results hypothesis suggests that complex neural computations underlying cortical processing could be temporally discrete. Moreover, we propose that cortical processing is produced by the computation of discrete spatio-temporal functional units. But what could be the neuronal elements underlining this computation? The cerebral cortex is composed of many types of neurons. Although all of them play a key role in cortical processing, our hypothesis suggests that there must be a specific type of inhibitory cell that may be implicated in the creation of a spatio-temporal structure supporting discrete cortical computation. We propose that FS interneuron may be a key element in our hypothesis providing the basis for this computation. These cells forming a synchronized spatially distributed cortical network may impose a temporal spike restriction in PCs creating functionally coupled units of computation. Their rhythmic activity may create a sequence of spatio-temporal functional units (Discrete Results), discretizing the information processing. In sum, we propose that they are able to integrate the spatial and temporal dimension of cortical computation.

FS cells (Kawaguchi and Kubota, 1997) are the largest population of interneurons in the neocortex. They play a key role as pacemakers for oscillations (Whittington et al., 1995; Traub et al., 1996) and in shaping multineuronal activity (Cardin et al., 2009). However, it is still unclear how these cells functionally contribute to the operations performed by the cortex.

It is known that they form dense matrices covering PCs (Packer and Yuste, 2011) extending a blanket of inhibition onto them (Karnani et al., 2014). They strategically innervate the axon initial segment (chandelier cells) or soma/proximal (basket cells) dendrites of PCs (Klausberger and Somogyi, 2008). They shape the precise timing and dynamic range of action potentials produced by PCs (Pouille and Scanziani, 2001; Cardin et al., 2009; Sohal et al., 2009; Li et al., 2015). They generate synchronized networks by mutual chemical and electrical connections (Galarreta and Hestrin, 1999; Gibson et al., 1999; Sohal et al., 2009). Accordingly, they fire in high synchrony (Jones et al., 2000) at high-frequency firing pattern without a significant spike adaptation (Kawaguchi and Kubota, 1997). They have narrow spike-waveform, fast kinetics (Atallah et al., 2012) and high synchronous release of GABA (Hefft and Jonas, 2005). Furthermore, they show broader tuning than other neurons (Kerlin et al., 2010; Hofer et al., 2011; Li et al., 2015). Thus, in accord with our proposal, these properties render them well suited for a structural role in cortical processing. Our hypothesis suggests that these cells create a temporal structure or scaffold (Temporal Structures of Spikes) providing alternating windows of no spiking (Silent Gaps) in the emergent Ensemble of PCs. Since different classes of FS cells have distinct properties in their temporal pattern of discharge (Gupta et al., 2000; Dehorter et al., 2015), it is then likely that they create diverse temporal restriction in PCs firing forming different Temporal Structures of Spikes. Moreover, we propose that different synchronized networks of FS interneurons create different sets of possible Ensembles. Experimental work supports this idea. Distinct clusters of FS interneurons have been identified in the cortex. For example, in the rat barrel cortex, one layer 4 FS interneuron type has an axonal domain strictly confined to a barrel (Koelbl et al., 2015).

Our hypothesis suggests that precise dynamic sequences of Discrete Results is the mechanism used by the cortex to extract, code, memorize and transmit neural information and that FS cells could play a key role in this discrete cortical processing. In agreement with that proposal, these cells are essential for perception, cognition, attention, memory and behavior (Isomura et al., 2009; Letzkus et al., 2011; Yizhar et al., 2011; Courtin et al., 2014; Hu et al., 2014; Kim et al., 2016). They are also implicated in plasticity and learning (Hensch, 2005; Yazaki-Sugiyama et al., 2009; Letzkus et al., 2011; Donato et al., 2013) and have been implicated in psychiatric disorders such as epilepsy and schizophrenia (Powell et al., 2003; Lewis et al., 2012). A prediction of our hypothesis would be that silencing of these neurons will disrupt normal cortical processing. Recently, it has been shown that silencing of these interneurons disrupts attentional processing (Kim et al., 2016). Furthermore, our hypothesis suggests that discrete cortical processing can be dynamically adjusted to meet the finest processing resolution depending on perceptual, task or attentional demands. We propose that increasing the number of Discrete Results per temporal unit allows resolution enhancement. Accordingly, experimental data show that perceptual coding and discrimination are improved by increased spiking of these cells (Lee et al., 2012). Moreover, in agreement with our proposal, increases in task difficulty and attentional requirements are accompanied by an enhancement of FS cells firing (Chen et al., 2008).

## Conclusion

There is increasing evidence that most neuronal activity in the cortex occurs in the form of coactive groups of cells defining neuronal ensembles. However, it is unclear what exactly an ensemble functionally means. These ensembles of neurons can form emergent functional units. In this Hypothesis and Theory article, we propose a new functional theoretical framework to explain the computational roles of these ensembles in cortical processing. We suggest that complex neural computations underlying cortical processing could be temporally discrete and that sensory information would need to be quantized to be computed by the cerebral cortex. Accordingly, we propose that cortical processing is produced by the computation of discrete spatio-temporal functional units that we have called Discrete Results. For example, perceptual integration, processing resolution and information coding can be now explained by our hypothesis.

The Discrete Result concept explains how complex neural computations underlying cortical processing could be temporally discrete. This novel concept has the ability to integrate the physiological and computational aspects of cortical processing defining the traditional idea of cells ensemble limiting their spatio-temporal dimension and differentiating their membership and relations between the members. Moreover, the Discrete Results hypothesis constitutes a conceptual advance with special relevance for neuroscience and computer sciences.

## Author Contributions

CC: conceived the hypothesis. CC and AN: conceptually developed and wrote this article.

## Funding

Work was supported by a grant from Ministerio de Economia y Competitividad (BFU2012-36107).

## Conflict of Interest Statement

The authors declare that the research was conducted in the absence of any commercial or financial relationships that could be construed as a potential conflict of interest.
